# Stability and Ultrafast Dynamics of Luminescent Biquinoxen-*Bis*-σ^H^-Adducts

**DOI:** 10.3390/molecules30204115

**Published:** 2025-10-16

**Authors:** Jonas Braun, Julia Leier, Mikhail Khorenko, Nicolas Leblanc, Christopher E. Anson, Wim Klopper, Claus Feldmann, Claudia Bizzarri, Andreas-Neil Unterreiner, Annie K. Powell

**Affiliations:** 1 Institute of Inorganic Chemistry (AOC), Karlsruhe Institute of Technology (KIT), Kaiserstraße 12, 76131 Karlsruhe, Germanychristopher.anson@kit.edu (C.E.A.); claus.feldmann@kit.edu (C.F.); 2 Institute of Nanotechnology (INT), Karlsruhe Institute of Technology (KIT), Kaiserstraße 12, 76131 Karlsruhe, Germany; 3 Institute for Quantum Materials and Technologies (IQMT), Karlsruhe Institute of Technology (KIT), Kaiserstraße 12, 76131 Karlsruhe, Germany; 4 Institute of Physical Chemistry (IPC), Karlsruhe Institute of Technology (KIT), Kaiserstraße 12, 76131 Karlsruhe, Germanywillem.klopper@kit.edu (W.K.); 5 Institute of Organic Chemistry (IOC), Karlsruhe Institute of Technology (KIT), Kaiserstraße 12, 76131 Karlsruhe, Germany; claudia.bizzarri@uniroma2.eu; 6 Department of Chemical Sciences and Technologies, University of Rome Tor Vergata, Via della Ricerca Scientifica, 00133 Rome, Italy

**Keywords:** biquinoxen, photoluminescence, femtosecond transient absorption spectroscopy, spectroelectrochemistry

## Abstract

We report the synthesis of two new biquinoxen-σ^H^-adducts (3,3′-diisopropoxy-4,4′-dimethyl-3,3′,4,4′-tetrahydro-2,2′-biquinoxaline (Mbqn-(O^i^Pr)_2_) (**1**) and 3,3′-bis(isopropylthio)-4,4′-dimethyl-3,3′,4,4′-tetrahydro-2,2′-biquinoxaline (Mbqn-(S^i^Pr)_2_) (**2**)) with the same molecular structure other than the exchange of two oxygen atoms with sulphur atoms. This enables us to directly compare the optical properties and stability of the compounds as a result of this substitution. For freshly prepared solutions of **1**, a fluorescence quantum yield of 97% is observed, whereas for **2**, the value is much lower at 7%. We furthermore note a decrease in quantum yields for solutions investigated after certain storage times, indicating a reactive channel. We note that this decomposition is much faster for solutions of **2** compared with compound **1**. For **1**, the decomposition likely proceeds to the biquinoxen dipseudobase via an equilibrium, whereas for **2** the decomposition product remains unidentified. The decomposition of **1** in particular was followed using ultrafast transient absorption spectroscopy, investigating the dynamics of the biquinoxen system after photoexcitation. Given the redox activity of biquinoxens, additionally the oxidation of the compounds was investigated using (spectro)electrochemistry.

## 1. Introduction

Molecules with multiple accessible redox states are important for many biological processes [1,2,3] and lend themselves to many applications such as (photo)catalytic systems [4,5,6], battery materials [7,8], and molecular machines [9], for example. Among such redox-active molecules, members of the viologen family (4,4′-disubstituted-bipyridinium salts) have gathered significant attention as a result of their unique electronic properties and the tunability given by the possibility for different residues [10,11,12,13].

The introduction of further heteroatoms to the viologen system led to the potential use of such molecules as ligands in coordination compounds akin to 2,2′-bipyridine-based ligands [14]. Using redox non-innocent molecules in conjunction with paramagnetic metal ions, for example, promotes magnetic interactions between the magnetic centres [15,16,17] and can make it possible to redox-switch the magnetic response of a system [18,19].

**Scheme 1:**
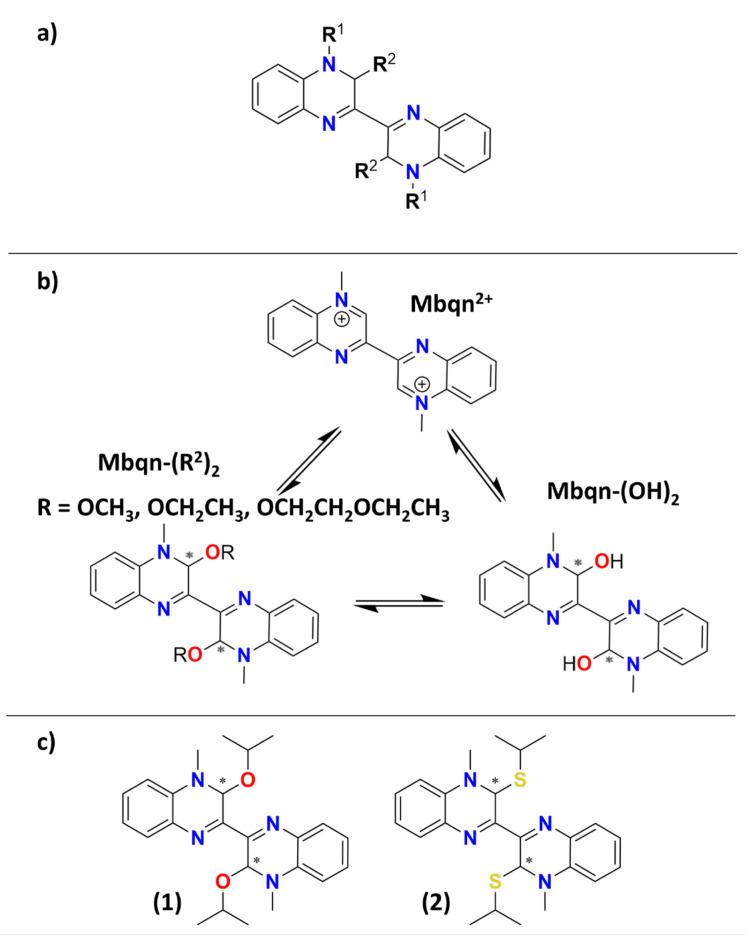
(**a**) General structure of the biquinoxen family; (**b**) reversible reactions between three forms of the methylbiquinoxen platform as described previously [20,21,22]; (**c**) new biquinoxen-*bis*-σ^H^-adducts reported here.

Inspired by this, some of us have previously reported the synthesis of the methylbiquinoxen system (Mbqn) (see Scheme 1a with R^1^ = Me and R^2^ = H) [20]. This system has not only been shown to possess switchable redox states but also to be capable of acting as a chelating ligand [20]. In a follow-up study, the system was expanded through the development of a new synthetic route to access additional R^1^-substituted biquinoxen derivatives (R^1^ = methyl, ethyl, propyl, and benzyl; R^2^ = H) [21]. The modifiability of the biquinoxen platform was furthermore accentuated through the discovery of an equilibrium between the methylbiquinoxen dication (Mbqn^2+^) with R^1^ = Me and R^2^ = H, the methylbiquinoxen dipseudobase (Mbqn-(OH)_2_) with R^1^ = Me and R^2^ = OH, and the methylbiquinoxen-bis-σ^H^-adducts (Mbqn-(OR)_2_) with R^1^ = Me and R^2^ = OCH_3_, OCH_2_CH_3_, or OCH_2_CH_2_OCH_2_CH_3_ (see Scheme 1b) [22]. In this study, the switchability between these forms, dependent on solvent and pH, as well as their optical properties, has been investigated. Whereas the dication exhibits cyan-coloured luminescence, the dipseudobase is non-luminescent, and the σ^H^-adducts (R^2^ = OCH_3_, OCH_2_CH_3_, OCH_2_CH_2_OCH_2_CH_3_) exhibit bright yellow emission [22].

To the best of our knowledge, no photophysical studies of the excited state properties of biquinoxens have been conducted within the appropriate time domain region of femtoseconds to nanoseconds. Conversely, experimental and theoretical work on intersystem crossing of quinoxalines suggests times of around 20 to 30 ps, with an accurate quantum yield for triplet state formation depending on the surrounding medium [23,24,25]. As we show, this differs significantly for biquinoxens; for example, the O-based compound exhibits a very high fluorescence quantum yield, indicating singlet state dynamics and reactivity.

Given the prospective use of the σ^H^-adduct dyes as sensitising ligands in coordination clusters, we here investigate the dynamics after electronic excitation as well as the stability of the biquinoxen compounds. Thus, we report the synthesis and optical properties of two new biquinoxen-*bis*-σ^H^-adducts (see Scheme 1c) and expand the family of compounds to sulphur-based σ^H^-adducts. This also gives us the opportunity to gauge the influence of oxygen vs. sulphur on the luminescence properties and dynamics after photoexcitation in structurally similar compounds. This constitutes the first attempt to understand the ultrafast processes that occur in biquinoxen-based molecules, aiming towards future application as a sensitising ligand in metal complexes.

## 2. Results

The structures of 3,3′-diisopropoxy-4,4′-dimethyl-3,3′,4,4′-tetrahydro-2,2′-biquinoxaline (Mbqn-(O^i^Pr)_2_) (**1**) and 3,3′-bis(isopropylthio)-4,4′-dimethyl-3,3′,4,4′-tetrahydro-2,2′-biquinoxaline (Mbqn-(S^i^Pr)_2_) (**2**) are shown in Scheme 1c. Both compounds can be obtained starting from the methylbiquinoxen dipseudobase (Mbqn-(OH)_2_). Whereas the synthesis of **1** follows the previously described procedure to obtain O-based σ^H^-adducts using 2-propanol as a solvent, compound **2** is obtained through the reaction of Mbqn-(OH)_2_ with 2-propanethiol in THF followed by evaporation of the solvent (see Section 3 for more details).

### 2.1. Crystallography

**Figure 1:**
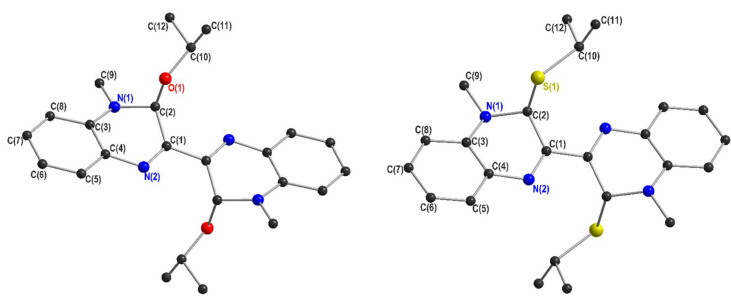
Molecular structures of **1** and **2** were obtained using single-crystal X-ray diffraction. Hydrogen atoms omitted for clarity.

Crystals suitable for X-ray diffraction were obtained for both compounds. Compound **1** crystallises in the triclinic space group *P*$\bar{1}$ with Z = 2, whereas compound **2** crystallises in the monoclinic space group *P*2_1_/*c* with Z = 2. The molecular structures of the two compounds are shown in Figure 1. Both centrosymmetric molecules show the same overall molecular structure with two essentially co-planar methylbiquinoxen halves with the O- and S-based residues pointed essentially perpendicular to this plane. The carbon atoms bonded to the residues (C2 and C2′) are bent out of the plane of the respective halves of the biquinoxen molecules. Whereas for compound **1**, C2 makes a distance of 0.114 Å to the plane of the phenyl ring (C3-C8), the corresponding distance in **2** is longer at 0.175 Å, indicating a larger distortion from planarity. This may contribute to a larger delocalisation and thus stabilisation effect in **1** compared to **2**.

### 2.2. Steady-State UV-Vis Absorption Spectroscopy

**Figure 2:**
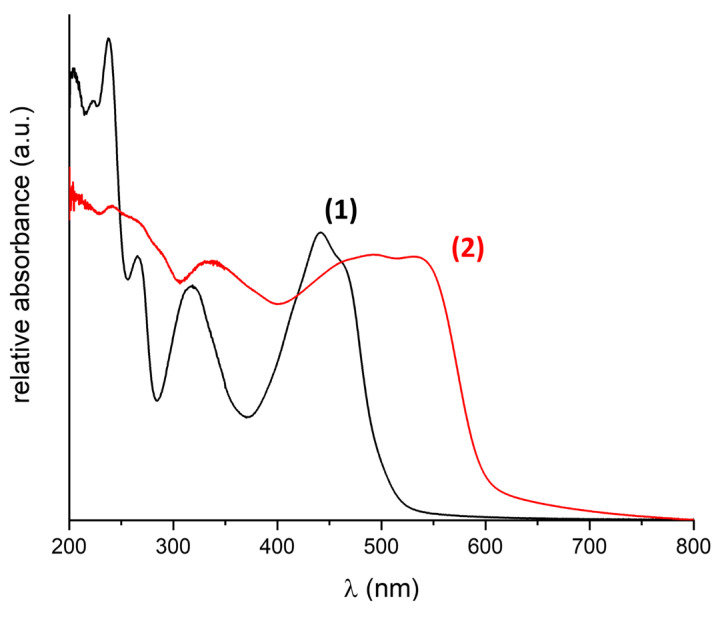
Solid-state absorption spectra of compounds **1** and **2**. Compound **2** is not only red-shifted but also shows a higher background contribution, likely resulting from scattering effects. These scattering effects can arise since the sample preparation method does not allow for control over particle size and sample thickness, for example [26].

The solid-state absorption spectra of compounds **1** and **2** are shown in Figure 2. Samples were prepared by submerging ground powder samples in mineral oil and pressing between two quartz plates, generating a homogenous layer of powder in oil. The orange-coloured compound **1** shows absorption bands in the UV region at 206, 223, 238, 265, and 316 nm and a coalesced band at 441/465 nm. This is in line with the spectra reported for the O-Me adduct reported previously [22]. The S-based compound **2** is red, which is reflected in the absorption spectrum. In the UV region, the two spectra show features at similar wavelengths, which can thus likely be attributed to the π-system, which remains unchanged. For compound **2**, the signal-to-noise ratio close to 200 nm is low, likely a result of scattering effects. The band, which was observed at 317 nm for **1**, however, is red-shifted in **2** by 20 nm, and the twin feature in the visible region is red-shifted by ca. 60 nm and broadened significantly, explaining the darker colour of the compound. Thus, these excitations are likely related to the residue of the methylbiquinoxen adducts.

**Figure 3:**
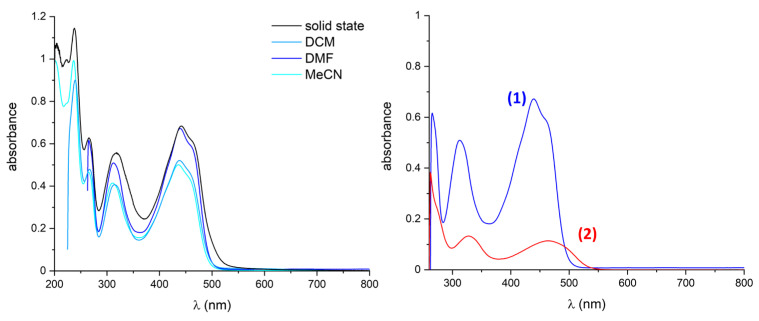
UV-Vis absorption spectra of compound **1** in solid state and different solvents (left); UV-Vis absorption spectra of DMF solutions of compounds **1** and **2** (right). All solution spectra were recorded from dilute 0.01 mM solutions.

In order to assess the behaviour of the biquinoxen dyes in solution, first the solubility was tested. The O-based compounds were readily soluble in MeCN, DCM, and DMF, as shown in Figure 3, in which the absorption spectra of solutions of **1** in these solvents are compared with the solid-state absorption spectrum. From these measurements, it can be concluded that the O-based adducts are rather stable in solution since the solid-state absorption spectrum can be reproduced excellently in the tested solvents (see Figure 3). Since **2** is essentially insoluble in all tested solvents but DMF, DMF was used for all subsequent solution studies in order to compare the solution state behaviour and gauge the effect of a sulphur atom in place of the oxygen atom. The absorption spectra of 0.01 mM solutions of **1** and **2** in DMF are compared in Figure 3.

As a result of the solvent cut-off of DMF, the solution state spectra are compared from 260 nm. Similarly to the behaviour in the solid state, the feature in the spectrum of **2** is red-shifted from 313 to 328 nm and from 440/460 nm in **1** to 465 nm in **2**. Thus, we can conclude that the transitions observed in UV-Vis spectroscopy appear to be comparable between the two compounds, but also that the inclusion of an S-atom instead of the O-atom reduces the energy gap between the ground and excited states associated with this part of the molecule. Furthermore, from the difference in extinction coefficient, one can conclude that the efficiency of the excitation in compound **2** is significantly reduced.

### 2.3. Quantum Chemical Calculations

The red-shift in absorption wavelength as well as the relative intensity of the absorption with respect to the oscillator strengths (see Supplementary Materials, Table S7 for oscillator strengths) from **1** to **2** are qualitatively reproduced by quantum chemical calculations for which the equilibrium geometries of the ground states (*S*_0_) of the two compounds were optimised by means of density-functional-theory (DFT) calculations using the PBE0 [27] and *ω*B97X [28] exchange–correlation functionals and the def2-SVPD basis set of Gaussian-type atomic orbitals [29].

The TURBOMOLE programme package was used for all calculations [30]. The results are consistent with the experimentally observed behaviour and are summarised in Tables S3–S8.

### 2.4. Emission Spectroscopy

Compound **1** shows orange luminescence under irradiation with a UV lamp operating at 365 nm. Thus, the solid-state emission spectrum of **1** was recorded and is shown in Figure S1. After excitation at 365 nm, the wavelength of the emission maximum was determined as 615 nm with an absolute quantum yield of 13%, comparable to those of other previously reported biquinoxen adducts [22]. For the S-based adduct **2**, no solid-state emission was observed.

**Figure 4:**
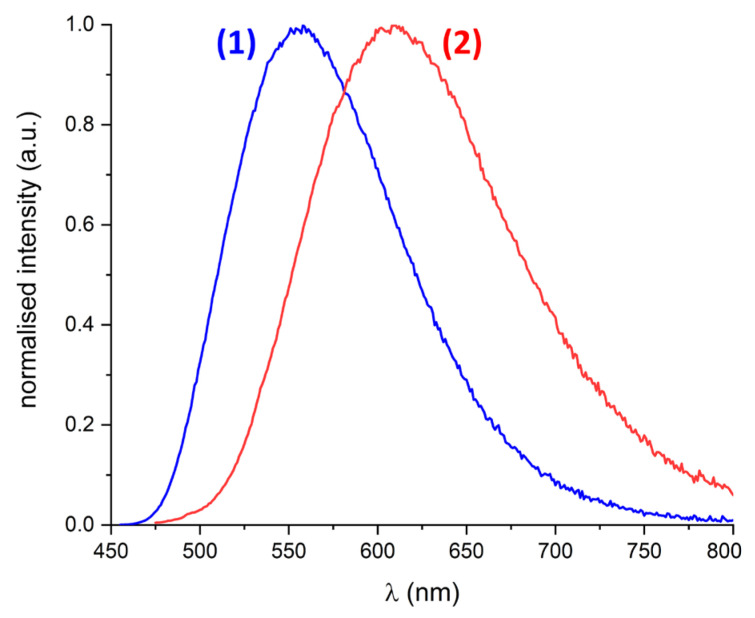
Emission spectra of 0.01 mM DMF solutions of **1** and **2** showing the tuning of luminescence wavelength through the exchange of an O-atom by an S-atom.

In Figure 4, the emission spectra of 0.01 mM DMF solutions of **1** and **2** are compared, and the corresponding emission maxima, lifetimes, quantum yields, and Stokes shifts are listed in Table 1. From these emission spectra, we can conclude that it is indeed possible to tune the emission wavelength of biquinoxen dyes utilising different substituents. In the present case, the emission wavelength could be varied by 52 nm between 558 nm in **1** and 610 nm in **2**.

The luminescence decay of compound **1** can be fitted using a single exponential, whereas compound **2** shows biexponential decay. Both compounds share a long-lived component with lifetimes close to 7 ns. For compound **2**, this component has an amplitude of 37%, while the remaining 63% belongs to a shorter-lived component with a lifetime of 0.4 ns. For compound **1**, this means that the lifetime is close to that observed for the literature-known O–Me adduct. In terms of quantum yield, a DMF solution of **1** with 97% significantly outperforms the O–Me adduct in MeCN, which has a reported value of 40%. A possible reason for this will be given in the discussion about the stability in solution below. Whereas the quantum yield of compound **1** with 97% is close to 100%, it is only 7% for **2**. The values of the calculated vertical excitation energies to the first excited singlet state calculated on the TD-DFT level reproduce the experiment well (see Table S4).

### 2.5. Quantum Yields and Equilibrium in Solution

When irradiating solutions of compound **1** stored for extended periods of time, it could be observed that the intensity of the fluorescence significantly decreases. In order to assess this behaviour and follow the decrease in quantum yield after the initial 97% measured for a freshly prepared solution, the quantum yield was successively measured after 2, 3, 5 and 24 h as well as after 2 and 3 days. The results are summarised in Table 2. Within a day, the quantum yield reduces from almost 100% to below 1%.

A possible explanation for this could be the existence of an equilibrium between the methylbiquinoxen-σ^H^-adduct and the methylbiquinoxen dipseudobase (Mbqn-(OH)_2_) starting material as described in reference [22] and shown in Scheme 1b. The two compounds and other forms that were identified to be present in the equilibrium can be distinguished by their absorption spectra and luminescence. In the case of **1** and the starting material Mbqn-(OH)_2_, the absorption spectra are identical, but the σ^H^-adduct shows bright luminescence, whereas the dipseudobase is non-emissive. Indeed, a ^1^H-NMR decomposition study of **1** in DMF-d_7_, during which several NMR spectra were recorded starting immediately after dissolution of the compound until 26 h thereafter, reveals the significant formation of several biquinoxen species, including the starting material Mbqn-(OH)_2_ after 1–2 h. Other forms of the compound that are potential candidates for species formed in solution, as well as more details on the proton NMR study, are shown in the SI (Figures S6–S12 and S14) [22].

Thus, the fact that the absorption spectrum of a DMF solution of **1** remains unchanged after multiple days while the emission intensity decreases significantly suggests that the equilibrium lies on the side of the dipseudobase in DMF solution [22].

For compound **2**, the decomposition in solution is substantially faster. A measurement of the quantum yield found that the yield reduces from 7% in the freshly prepared solution to 0.2% after 1 day. This can be rationalised in terms of the much lower quantum yield of freshly prepared solutions of **2**, which suggests that non-emissive (e.g., reactive) channels are favoured in this compound. The red-shifted absorption spectrum after 1 day, in combination with a quantum yield well below 1%, furthermore suggests the formation of the mono-σ^H^-adduct cation. This is feasible given that the S-based residue cannot react back to the dipseudobase.

### 2.6. Transient Absorption Spectroscopy

**Figure 5:**
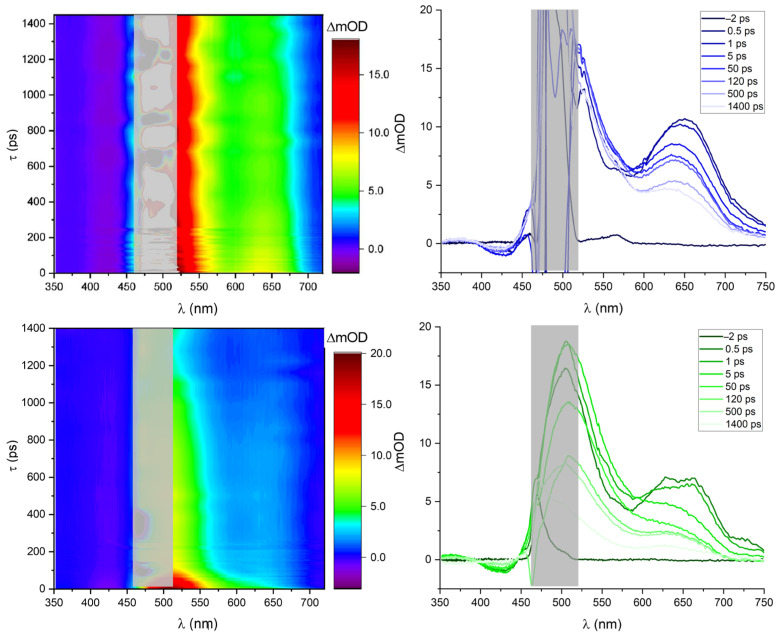
Contour plot (top left) and transient absorption spectra (top right) of a freshly prepared solution of compound **1** after excitation at 490 nm with delay times up to 1400 ps. Contour plot (bottom left) and transient absorption spectra (bottom right) of an aged solution of **1**. The grey bar hides scattering effects as a result of the 490 nm pump pulse.

In order to understand the ultrafast dynamics of the luminescent methylbiquinoxen σ^H^-adducts, transient absorption spectroscopic measurements were performed on DMF solutions of compound **1**. A freshly prepared solution of **1** was excited at 490 nm, and the response was probed using a white light continuum between 350 and 750 nm (see Figure 5, top).

We observe a dominant excited state absorption (ESA) band above 450 nm to the end of our spectral measurement window at 750 nm. At higher energies around 420 nm, a small negative signal is observed, which, when taking the steady-state absorption spectra into account, can be assigned to ground state bleaching (GSB). The isosbestic point between the GSB and ESA bands suggests that both dynamics originate from the same state. Both dynamics have lifetimes > 2 ns in agreement with the emission lifetimes determined for **1**. The stimulated emission (SE) is not detected as a negative response due to overlap with the dominant ESA band but can be seen as a local minimum at ca. 550 nm.

Excitation into higher excited states was investigated using a 317 nm pump pulse, which resulted in similar dynamics (see Figure S2).

In order to investigate the effect of the equilibrium in solution, the sample, now with a fluorescence quantum yield of < 1%, was remeasured after a few days (see Figure 5, bottom). As evident from the contour plots and absorption spectra, the dynamics between 600 and 750 nm are much shorter-lived and essentially vanish after ca. 100 ps. This change in the lifetime of the excited state dynamics is another indication of the presence of an equilibrium with a different but similar compound, such as the dipseudobase in solution after multiple hours.

**Figure 6:**
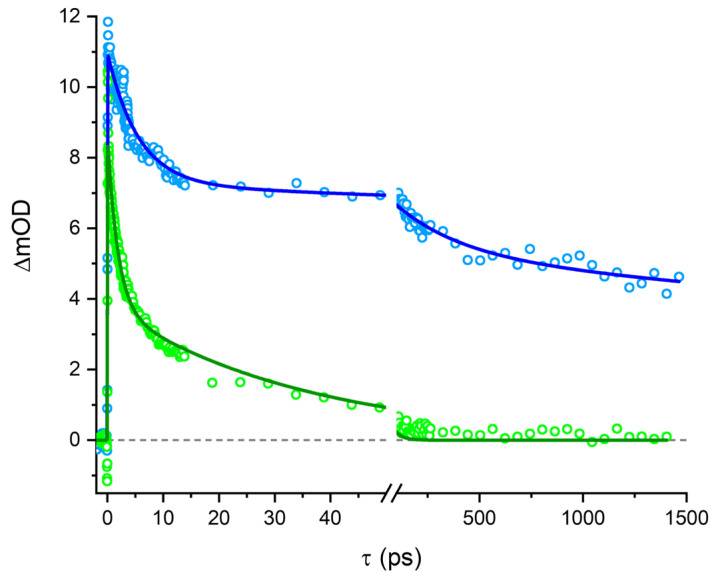
Single transient spectra at a probe wavelength of 650 nm of a fresh (blue) and an aged solution of **1** in DMF (green), excited at 490 nm.

These findings can further be corroborated by performing single transient analyses. The analyses for the single transients of both the fresh and the aged samples at **1** at 650 nm are shown in Figure 6. The ageing of the sample can easily be seen from the long-time constant τ_long_, which is multiple ns in the fresh solution and 35 ps in the aged sample. It can furthermore be seen that the long-lived component at 650 nm present in freshly dissolved Mbqn-(O^i^Pr)_2_ is no longer present once the solution has been stored for more than 2 days. Further single-wavelength analyses are shown in Figure S3. Global analysis is not feasible here since hypochromic shifts prevented an analysis using a simple ansatz of sums of exponential functions.

**Figure 7:**
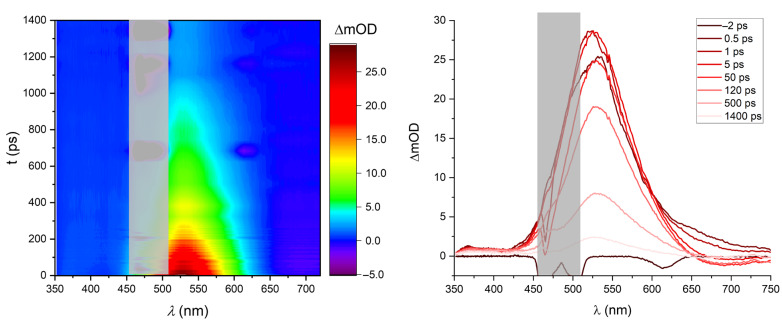
Contour plot (left) and transient spectra (right) of a freshly prepared solution of compound **2** in DMF after 490 nm excitation.

A fresh solution of **2** was subjected to the same transient spectroscopy experiment (see Figure 7). In contrast to the transient dynamics in compound **1**, here no GSB is observed at the position of the steady-state absorption band since it is overlapped with the dominant ESA band. However, the ESA is less pronounced than in **1**, which leads to the observation of SE at wavelengths > 660 nm. Although generally shorter-lived, the dynamics in **2** contain a long-lived component, qualitatively in agreement with the luminescence lifetimes shown above.

### 2.7. Oxidation of Mbqn-(O^i^Pr)_2_

Considering the redox-active nature of the biquinoxen platform, the chemical oxidation of **1** in DMF upon addition of H_2_O_2_ was tested, and an immediate colour change from bright yellow to orange/red was observed. In order to investigate this behaviour further, spectroelectrochemical experiments were performed on a freshly prepared, argon-flushed DMF solution of **1**. As shown in Figure S4, two irreversible oxidation processes can be observed at an anodic potential of 0.66 and 0.84 V vs. Fc/Fc^+^.

**Figure 8:**
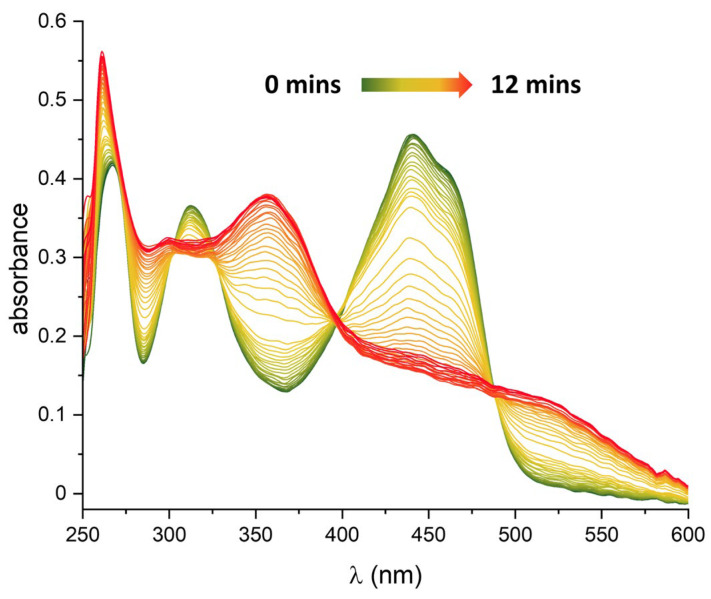
Spectroelectrochemical measurement of a freshly prepared solution of **1** in DMF using TBAPF_6_ as electrolyte. A voltage of 0.7 V vs. Fc/Fc^+^ was applied, and UV-Vis absorption spectra were measured every 15 s over the timeframe of 12 min (starting from green to red).

The abovementioned colour change was followed by applying a voltage of 0.7 V vs. Fc/Fc^+^ and recording a UV-Vis spectrum every 15 s (see Figure 8). The intense bands at 440/460 nm and 315 nm disappear. Simultaneously, at least four distinct new bands arise (520, 355, 300, and 265 nm). Several isosbestic points indicate a direct conversion from the original compound to the product. Similar behaviour was observed for compound **2**. The irreversible oxidation processes occur at 0.47 and 0.75 V vs. Fc/Fc^+^. Spectral changes upon oxidation are reported in Figure S5, where the main bands centred at 320 nm and 475 nm decrease their intensity while two other broader bands appear at 370 nm and 545 nm.

Despite our efforts, we were not able to determine the structure of the oxidised species that is formed.

## 3. Materials and Methods

**Crystallography.** Data were measured on Stoe STADIVARI diffractometers (STOE & Cie GmbH, Darmstadt, Germany) equipped with MetalJet2 liquid Ga rotating anode (1) or GeniX 3D HF Mo-microfocus (2) sources. Data were corrected for absorption, and the structures were solved by dual-space direct methods with intrinsic phasing (SHELXT [32]) followed by full-matrix least-squares refinement against F^2^ (SHELXL-2019 [33]) within the Olex2 v1.5 platform (Regensburg, Germany) [34]. All non-H atoms were assigned anisotropic temperature factors. For **1**, all H-atoms were freely refined (coordinates and Uiso), while for **2**, H-atoms were placed in calculated positions with a riding model.

From the unit cell parameters, it can be seen that the two unit cells are closely related. The unit cell for **2** can be obtained by adjusting the α and β angles for the cell of **1** each to 90° and reducing γ from 111° to 94°, followed by the necessary permutation of the axes for the now monoclinic cell. The monoclinic symmetry operations in the cell of **2** can also be seen as approximate relationships between the independent half-molecules in **1**. This apparent phase transformation may well be the reason for the poor crystal quality found for **2**, although the crystal structure is completely adequate to show that **2** is isostructural to **1**.

Full crystallographic data and details of the structural determinations for the structures in this paper have been deposited with the Cambridge Crystallographic Data Centre as supplementary publication nos. CCDC 2466946 and 2466947. Copies of the data can be obtained, free of charge, from https://www.ccdc.cam.ac.uk/structures/ (accessed on 3 July 2025).

**Photoluminescence spectroscopy.** Excitation and emission spectra were recorded using a photoluminescence spectrometer, Horiba Jobin Yvon Spex Fluorolog 3 (Oberursel, Germany), equipped with a 450 W Xenon lamp, a double monochromator for excitation and emission, an integrating sphere (Ulbricht sphere), and a photomultiplier as the detector. The determination of the quantum yield was performed according to Friend et al. [35]. First of all, the diffuse reflection of the sample was determined under excitation conditions. Thereafter, the emission was measured at this excitation wavelength. Integration over the reflected and emitted photons with an Ulbricht sphere results in the absolute quantum yield. Corrections were made regarding the spectral power of the excitation source, the reflection behaviour of the Ulbricht sphere, and the sensitivity of the detector.

**Transient Absorption Spectroscopy.** The experimental setup has been described in detail previously [36,37]. Modifications and a brief summary of the present experiments are outlined in the following. To obtain time-resolved spectra in the UV-Vis range, a small part (2–3 μJ) of an 800 nm laser output (Astrella, Coherent, Dieburg, Germany), 7 mJ, 35 fs, repetition rate 1 kHz) was propagated to a computer-controlled translation stage (maximum delay ∼1.8 ns, Thorlabs, Bergkirchen, Germany) and focused into a movable 2 mm CaF_2_ crystal (nortus Optronic GmbH, Wörth am Rhein, Germany) to generate a white-light continuum between 350 and 720 nm. A reference beam and the probe’s white light were refracted by a fused silica prism and recorded by two CCD cameras (Series 3000, Si Photodetector, Entwicklungsbüro Stresing, Berlin, Germany). The calibration of the white light was achieved with eight interference filters (FWHM = 10 ± 2 nm, Thorlabs, Bergkirchen, Germany) in 50 nm steps from 350 to 700 nm, resulting in a calibration accuracy of 2–3 nm. The pump wavelengths at 490 and 317 nm (pulse duration < 50 fs) were obtained from a noncollinear optical parametric amplifier (NOPA, Clark-MXR Inc., Dexter, MI, USA) output (490 nm as a fundamental beam and 317 nm after frequency-doubling of a 634 nm NOPA fundamental in a BBO crystal). The spot size of the pump beam in the sample was about 200 μm, ca. twice the spot size of the white light. Excitation energies were 250–400 nJ per pulse. Every second pump pulse was blocked with an optical chopper (Thorlabs, Bergkirchen, Germany), resulting in spectra indicating the pump-induced change in the optical density (ΔmOD). Data were collected and processed with an in-house written LabVIEW programme (National Instruments, Munich, Germany). All transient absorption spectra were measured in solution at room temperature in fused silica cuvettes (Hellma, München, Germany and Starna, Pfungstadt, Germany) with 1 mm optical path lengths.

**Quantum Chemical Calculations.** During the geometry optimizations, the convergence threshold for the energy was set to 10^−9^ *E*_h_ and the corresponding threshold for the nuclear gradient to 10^−6^ *E*_h_/*a*_0_ (the energy threshold in the self-consistent-field calculations was set to 10^−10^ *E*_h_). TURBOMOLE’s grid 5 was used, and its derivatives were taken into account when computing the analytical nuclear gradients. The optimised ground-state equilibrium geometries exhibited *C*_i_ point-group symmetry, and harmonic vibrational frequencies were computed to verify that the geometries represent minima on the potential energy hypersurfaces.

The equilibrium geometries of the first excited singlet states (*S*_1_) of both compounds were optimised by means of time-dependent DFT (TD-DFT) calculations using the PBE0 and *ω*B97X exchange–correlation functionals and the def2-SVPD basis set of Gaussian-type atomic orbitals. These first excited singlet states transform according to the irreducible representation *A*_u_ of the symmetry point group *C*_i_. The energy threshold for the excited-state energy was set to 10^−6^ *E*_h_ (rpaconv = 6).

Single-point calculations of singlet excited-state energies and ordinary photoabsorption spectra were performed using the Bethe-Salpeter equation (BSE) formalism as implemented in TURBOMOLE [38]. These BSE calculations were also carried out in the correlation-kernel-augmented variant (cBSE) [39]. The quasiparticle energies required by the BSE and cBSE methods were obtained at the eigenvalue-consistent *GW* level (ev*GW*) using the contour deformation technique as described in [40], using 128 quadrature points for the numerical integration along the imaginary axis. About the Fermi level, two unoccupied and four occupied levels were accounted for in the ev*GW* calculations with the PBE0 functional. When using the *ω*B97X functional, two unoccupied and two occupied levels were considered. All of the ev*GW*, BSE, and cBSE calculations were performed in the resolution of the identity approximation using the optimised def-SVPD auxiliary basis set of Hellweg and Rappoport [41]. The excited-state energy threshold was set to rpaconv = 6, and the damping parameter was set to *δ* = 0 [40].

**Electrochemistry and Spectroelectrochemistry.** Electrochemical data were acquired with a Gamry Interface 1010 B (Warminster, UK), equipped with a three-electrode cell using a Pt disc as the working electrode, a platinum wire as the counter electrode, and a silver wire as a quasi-reference electrode, employing the ferrocene/ferrocenium couple as an internal standard. Spectroelectrochemical experiments were performed in a special cuvette, with an optical path of 1.5 mm, using an optically transparent working electrode, such as a platinum grid. The spectra were recorded using an ALS SEC 2020 wide wavelength range spectrometer (all Tokyo, Japan).

**^1^H-NMR.** Spectra were recorded on a Bruker Avance Neo (400 MHz, Ettlingen, Germany).

**Synthesis.** All syntheses, unless indicated otherwise, were performed in air. The methylbiquinoxen dipseudobase (Mbqn-(OH)_2_) was produced after a literature procedure [22]. All other reagents were obtained from commercial sources (Sigma Aldrich Chemie GmbH, Taufkirchen, Germany; abcr, Karlsruhe, Germany) and used without further purification.

**3,3′-diisopropoxy-4,4′-dimethyl-3,3′,4,4′-tetrahydro-2,2′-biquinoxaline (methylbiquinoxen *iso*-propyl-adduct, Mbqn-(O*^i^*Pr)_2_) (1)**

50.0 mg of Mbqn-(OH)_2_ (0.155 mmol) were dissolved in hot 2-propanol (30 mL) and stirred for 30 min. The hot solution was filtered, and the filtrate was left undisturbed for slow evaporation. The product was obtained after 2 days as bright orange crystals in a yield of 83%.

**^1^H-NMR (500 MHz, DMF-d_7_): δ/ppm** = 7.55–7.53 (m, 2H, CH_AR_); 7.38–7.35 (m, 2H, CH_AR_); 7.11–7.09 (m, 2H, CH_AR_); 7.02–6.98 (m, 2H, CH_AR_); 6.27 (s, 2H, CH_AR_); 4.12 (hept, J = 6.1 Hz, 2H, CH); 3.44 (s, 6H, CH_3_); 1.08 (d, J = 6.1 Hz, 6H, CH_3_); 0.95 (d, J = 6.1 Hz, 6H, CH_3_). **IR (4000–400 cm^−1^):** 3050 (b, w); 2970 (m); 2929 (w), 2865 (w), 1677 (b, w); 1603 (m); 1554 (m); 1492 (s); 1449 (m); 1428 (w);1371 (m); 1330 (m); 1309 (s); 1275 (m); 1221 (s); 1162 (m); 1137 (m); 1116 (m); 1098 (m); 1051 (w); 1032 (w); 985 (s); 973 (s); 911 (s); 874 (m); 800 (m); 753 (s); 739 (s), 710 (m); 636 (w); 587 (w); 562 (m); 530 (w); 519 (w); 478 (m); 435 (w); 419 (w); 404 (w). **C/H/N experiment% (calculated%):** C: 70.67 (70.91), H: 7.32 (7.44), N: 13.89 (13.78).

**Crystal Data** for C_24_H_30_N_4_O_2_ (*M* =406.52 g/mol): triclinic, space group *p*-1 (no. 2), *a* = 9.2870(3) Å, *b* = 10.6054(3) Å, *c* = 11.8298(3) Å, *α* = 98.896(2)°, *β* = 90.332(2)°, *γ* = 110.925(2)°, *V* = 1072.86(6) Å3, *Z* = 2, *T* = 180(2) K, μ(GaKα) = 0.417 mm^−1^, *Dcalc* = 1.258 g/cm^3^, 15238 reflections measured (6.594° ≤ 2Θ ≤ 128.41°), 5211 unique (*R*int = 0.0144, Rsigma = 0.0176) which were used in all calculations. The final *R*1 was 0.0384 (I > 2σ(I)), and *wR*2 was 0.1048 (all data).

**3,3′-bis(isopropylthio)-4,4′-dimethyl-3,3′,4,4′-tetrahydro-2,2′-biquinoxaline (methylbiquinoxen *iso*-thio-propyl-adduct (Mbqn-(S*^i^*Pr)_2_) (2)**

A total of 50.0 mg of Mbqn-(OH)_2_ (0.155 mmol) was dissolved in hot THF (100 mL), and an excess of 2-propanthiol (6 mL, 63.8 mmol) was added. The mixture was stirred for 30 min at 60 °C before hot filtration and leaving the filtrate undisturbed to evaporate to dryness. The resulting solid was taken up in acetone and Et_2_O, filtered, and dried in air. The product was obtained as red crystals in a yield of 58%.

**IR (4000–400 cm^−1^):** 3060 (w), 3040 (w), 2955 (m), 2922 (m), 2861 (m), 1603 (m), 1554 (m), 1486 (m), 1449 (m), 1426 (w), 1375 (m), 1324 (m), 1303 (m), 1271 (m), 1242 (w), 1223 (m), 1197 (m), 1160 (m), 1123 (w), 1114 (w), 1096 (m), 1053 (w), 1034 (m), 965 (m), 930 (m), 872 (w), 817 (w), 754 (m), 741 (s), 712 (s), 683 (s), 632 (m), 587 (m), 550 (s), 524 (m), 480 (m), 472 (m), 445 (m), 423 (m). **C/H/N experiment% (calculated%):** C: 65.62 (65.71), H: 6.80 (6.89), N: 12.93 (12.77), S: 14.91 (14.62).

**Crystal Data** for C_24_H_30_N_4_S_2_ (*M* =438.64 g/mol): monoclinic, space group P2_1_/c (no. 14), *a* = 10.6835(11) Å, *b* = 11.7694(12) Å, *c* = 9.3090(9) Å, *β* = 94.629(8)°, *V* = 1166.7(2) Å^3^, *Z* = 2, *T* = 180 K, μ(Mo Kα) = 0.246 mm^−1^, *Dcalc* = 1.249 g/cm^3^, 9964 reflections measured (3.824° ≤ 2Θ ≤ 69.114°), 10229 unique (*R*_int_ = 0.1207, R_sigma_ = 0.1235) which were used in all calculations. The final *R*_1_ was 0.1377 (I > 4u(I)), and *wR*_2_ was 0.3828 (all data).

## 4. Conclusions

We investigated two novel biquinoxen-σ^H^-adducts, which show significant photophysical properties and redox activity. Compound **1** shows quantum yields close to 100% in freshly prepared DMF solutions. However, the behaviour in DMF solution of both compounds is characterised by their lability. To summarise the decomposition of **1** in DMF solution, the process can be followed using multiple experimental techniques. In the fresh solution, the fluorescence quantum yield is 97% (see Table 1). The ^1^H-NMR spectrum shows a clean compound (see Figures S6, S7 and S14), and the lifetime of the excited state dynamics exceeds the measurement window of the transient absorption spectroscopy setup (Figure 5). We observe significant decomposition within the first 2 h after dissolution, after which the ^1^H-NMR clearly shows the presence of several biquinoxen species (see Figures S8–S12), and the fluorescence quantum yield has decreased to ca. 64% (Table 2). After 24 h, the QY has fallen to less than 1% (Table 2), the original compound **1** can no longer be detected by NMR in significant amounts (Figures S12 and S14), and the transient response of the solution shows much shorter-lived dynamics (Figure 5). Compound **2** was shown to be even more prone to decomposition into an undefined product. Furthermore, in spectroelectrochemical measurements, we showed an irreversible oxidation that results in a colour change in the solution. The absorption spectrum of the electrochemically oxidised species furthermore indicates the likely oxidation of solid samples of **1** that are stored in air for many weeks. Future studies should be aimed at investigating the possible stabilisation of the biquinoxen-σ^H^-adducts as potential sensitising ligands in coordination chemistry.

## Figures and Tables

**Table 1:** Absorption and emission maxima, Stokes shifts, lifetimes, and corresponding quantum yields of 0.01 mM DMF solutions of **1** and **2**.

	λ_max,abs_ (nm)	λ_max,em_ (nm)	Stokes Shift (cm^−1^)	τ_1_ (ns)	τ_2_ (ns)	QY_1_
**1**	439	558	4858	6.9	-	0.97
**2**	469	610	4929	0.4	6.7	0.07

**Table 2:** Quantum yields obtained as relative quantum yields against a standard of [Ru(bpy)_3_]Cl_2_ [31] after storage times of the DMF solution of **1** between 2 h and 1 day.

Storage Time of Solution	QY
0 h ^1^	0.97
2 h	0.643
3 h	0.517
5 h	0.469
1 d	0.062

^1^ absolute quantum yields obtained in the abovementioned measurement of a freshly prepared sample of **1**.

## Data Availability

Full crystallographic data and details of the structural determinations for the structures in this paper have been deposited with the Cambridge Crystallographic Data Centre as supplementary publication nos. CCDC 2466946 and 2466947. Copies of the data can be obtained, free of charge, from https://www.ccdc.cam.ac.uk/structures/ (accessed on 3 July 2025). Further experimental data can be made available by the corresponding authors upon request.

## Supplementary Materials

The following supporting information can be downloaded at https://www.mdpi.com/article/10.3390/molecules30204115/s1: Figure S1: Solid state emission and excitation spectra of **1**. The peak at ca. 520 nm in the excitation spectrum indicates oxidation of **1** in line with the spectroelectrochemical data reported in the main text. Figure S2: Transient absorption spectra of a fresh solution of **1** after excitation at 317 nm. Figure S3: Single transient analyses of a fresh and an aged solution of **1**. Figure S4: Cyclic voltammetry of **1** and **2** (left and right) in DMF with 0.1 M TBAPF_6_ as electrolyte, revealing the position of the first oxidation, which is used for the spectroelectrochemical measurements. Figure S5: Spectroelectrochemical measurements of compound **2** at a voltage of 0.7 V vs. Fc/Fc^+^ reveal the interconversion of the pristine compound (green) to an oxidised species (red) as suggested by the isosbestic points. Table S1: Crystal data.; Table S2: Selected Bond Lengths (Å) for 1 and 2.; Table S3: C-C and C-N bond lengths as obtained in the def2-SVPD basis set. Table S4: Vertical and adiabatic excitation energies of the first excited singlet state (S_1_) as obtained at the TD-DFT level. Table S5: Vertical and adiabatic excitation energies of the first excited singlet state (S_1_) as obtained using the BSE formalism. Table S6: Vertical and adiabatic excitation energies of the first excited singlet state (S_1_) as obtained at the cBSE level. Table S7: Oscillator strengths f_OSC_ as obtained at the TD-DFT level. Table S8: Most important pair of hole and particle natural transition orbitals (NTOs) as obtained at the BSE level with the PBE0 functional in the def2-SVPD basis set. The NTOs are plotted with an isosurface value of ±0.025 $a_{0}^{-3/2}$. The weight of the pair is given in %. Reference [22] has been cited in the Supplementary Materials.
