# Supplementary material for: Stability and Ultrafast Dynamics of Luminescent Biquinoxen-Bis-σH-Adducts
Source: Molecules. 2025 Oct 16;30(20):4115. doi: 10.3390/molecules30204115 (PMC12566332; doi:10.3390/molecules30204115)
Supplement: Supplementary file 1 [file molecules-30-04115-s001.zip › molecules-3769513-supplementary.pdf]

Stability and Ultrafast Dynamics of Luminescent Biquinoxen-*bis*-σ^H-Adducts

Jonas Braun,*[a,b,c] Julia Leier,[d] Mikhail Khorenko,[a] Nicolas Leblanc,[b] Christopher E. Anson,[a] Wim Klopper,[d] Claus Feldmann,[a] Claudia Bizzarri,[e,f] Andreas-Neil Unterreiner,*[d] Annie K. Powell*[a,b,c]

a) Institute of Inorganic Chemistry (AOC), Karlsruhe Institute of Technology (KIT), Kaiserstr. 12 76131 Karlsruhe, Germany.

b) Institute of Nanotechnology (INT), Karlsruhe Institute of Technology (KIT), Kaiserstr. 12 76131 Karlsruhe, Germany.

c) Institute for Quantum Materials and Technologies (IQMT), Karlsruhe Institute of Technology (KIT), Kaiserstr. 12 76131 Karlsruhe, Germany.

d) Institute of Physical Chemistry (IPC), Karlsruhe Institute of Technology (KIT), Kaiserstr. 12 76131 Karlsruhe, Germany.

e) Institute of Organic Chemistry (IOC), Karlsruhe Institute of Technology (KIT), Kaiserstr. 12 76131 Karlsruhe, Germany.

f) Department of Chemical Sciences and Technologies, University of Rome Tor Vergata, via della Ricerca Scientifica, 00133 Rome, Italy.

Corresponding author emails: jonas.braun2@kit.edu, andreas.unterreiner@kit.edu and annie.powell@kit.edu

Table of Contents

Crystallography 2
Quantum Chemical Calculations 4
Solid State Emission Spectroscopy 6
Transient Absorption Spectroscopy 7
(Spectro)electrochemistry 9
NMR 11

Crystallography

Table S1. Crystal data.

Compound	**1**	**2**
Formula	$C_{24}H_{30}N_4O_2$	$C_{24}H_{30}N_4S_2$
Formula weight	406.52	438.64
Crystal System	Triclinic	Monoclinic
Space Group	$P\bar{1}$	$P2_1/c$
a / Å	9.2870(3)	10.6835(11)
b / Å	10.6054(3)	11.7694(12)
c / Å	11.8298(3)	9.3090(9)
α/ °	98.896(2)	90
β/ °	90.332(2)	94.629(8)
γ/ °	110.925(2)	90
V / $Å^3$	1072.86(6)	1166.7(2)
Z	2	2
T / K	180	180
F(000)	436	468
D_c / Mg m^{-3}	1.258	1.249
λ / Å	1.34143	0.71073
μ / mm^{-1}	0.430	0.246
Data Measured	15232	5770
Unique Data	5210	2382
R_{int}	0.0144	0.1078
Data with I ≥ 2σ(I)	4249	1776
wR_2 (all data)	0.1013	0.3371
S (all data)	1.062	1.300
R_1 [I ≥ 2σ(I)]	0.0371	0.1274

Parameters/Restraints	392 / 0	139 / 0
Biggest diff. peak/hole / eÅ^{-3}	+0.296 / -0.189	+0.855 / -0.917
CCDC number	2466946	2466947

Table S2. Selected Bond Lengths (Å) for **1** and **2**.

	**1**	**2**
C1-C1′	1.4750(19)	1.471(8)
C1-C2	1.5097(14)	1.505(5)
C2-O1/S1	1.4461(13)	1.883(4)
C2-N1	1.4284(13)	1.432(5)
N1-C9	1.4523(15)	1.456(5)
N1-C3	1.3781(15)	1.380(5)
C3-C4	1.4141(16)	1.416(6)
C4-N2	1.3949(13)	1.402(5)
N2-C1	1.2924(14)	1.287(5)

Quantum Chemical Calculations

The methods are described in detail in the main text.

Table S3. C-C and C-N bond lengths as obtained in the def2-SVPD basis set.

	C-C (pm)				C-N (pm)			
	PBE0		ωB97x		PBE0		ωB97x	
	S_0	S_1	S_0	S_1	S_0	S_1	S_0	S_1
**1**	146.3	141.7	148.2	141.3	129.1	132.5	128.1	133.1
**2**	146.6	141.9	148.4	141.5	128.9	132.5	127.9	133.2

Table S4. Vertical and adiabatic excitation energies of the first excited singlet state (S_1) as obtained at the TD-DFT level.

	λ (nm) PBE0			λ (nm) ωB97x		
	S_0 absorption	adiabatic	S_1 emission	S_0 absorption	adiabatic	S_1 emission
**1**	430.8	476.2	536.1	348.1	409.8	487.7
**2**	465.4	510.6	563.8	365.0	422.4	508.6

Table S5. Vertical and adiabatic excitation energies of the first excited singlet state (S_1) as obtained using the BSE formalism.

	λ (nm) PBE0			λ (nm) ωB97x		
	S_0 absorption	adiabatic	S_1 emission	S_0 absorption	adiabatic	S_1 emission
**1**	416.4	488.9	552.3	379.2	445.8	539.5
**2**	443.1	509.1	562.0	402.4	466.9	574.5

Table S6. Vertical and adiabatic excitation energies of the first excited singlet state (S_1) as obtained at the cBSE level.

	λ (nm) PBE0		
	S_0 absorption	adiabatic	S_1 emission
**1**	401.8	469.5	527.7
**2**	426.8	489.6	538.3

Table S7. Oscillator strengths f_{OSC} as obtained at the TD-DFT level.

	f_{OSC} PBE0		f_{OSC} ωB97x	
	S_0 absorption	S_1 emission	S_0 absorption	S_1 emission
**1**	0.4989	0.4776	0.5652	0.6000
**2**	0.3160	0.3297	0.3921	0.4839

Table S8. Most important pair of hole and particle natural transition orbitals (NTOs) as obtained at the BSE level with the PBE0 functional in the def2-SVPD basis set. The NTOs are plotted with an isosurface value of ±0.025 $a_0^{-3/2}$. The weight of the pair is given in %.

	%	Hole NTO	Particle NTO
**1**	96.1	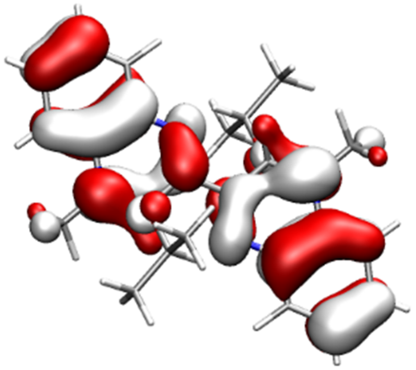	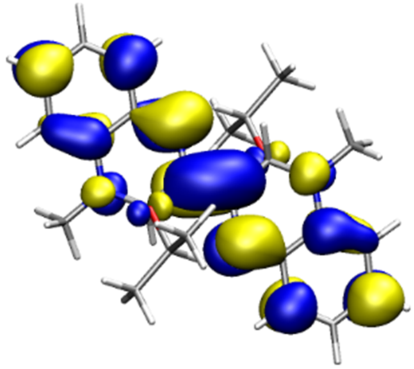
**2**	95.9	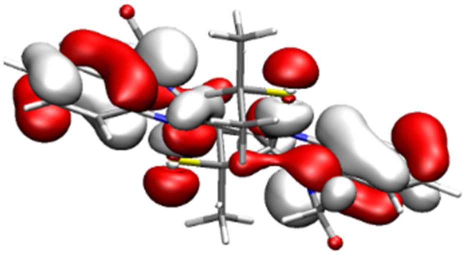	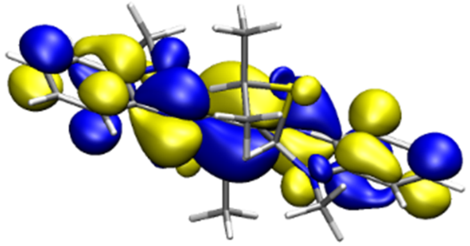

Solid State Emission Spectroscopy

The fluorescence of **1** in its solid state form was measured given the emission observed using a UV lamp. The absolute quantum yield was determined to be 0.13 which is in line with previously reported methylbiquinoxen-σ^H-adducts.[1] The band at 520 nm in the excitation spectrum which is not present in the corresponding solid state absorption spectrum suggests significant reabsorption of the compound during solid state luminescence measurements.

Compound **2** did not show emission in the solid state.

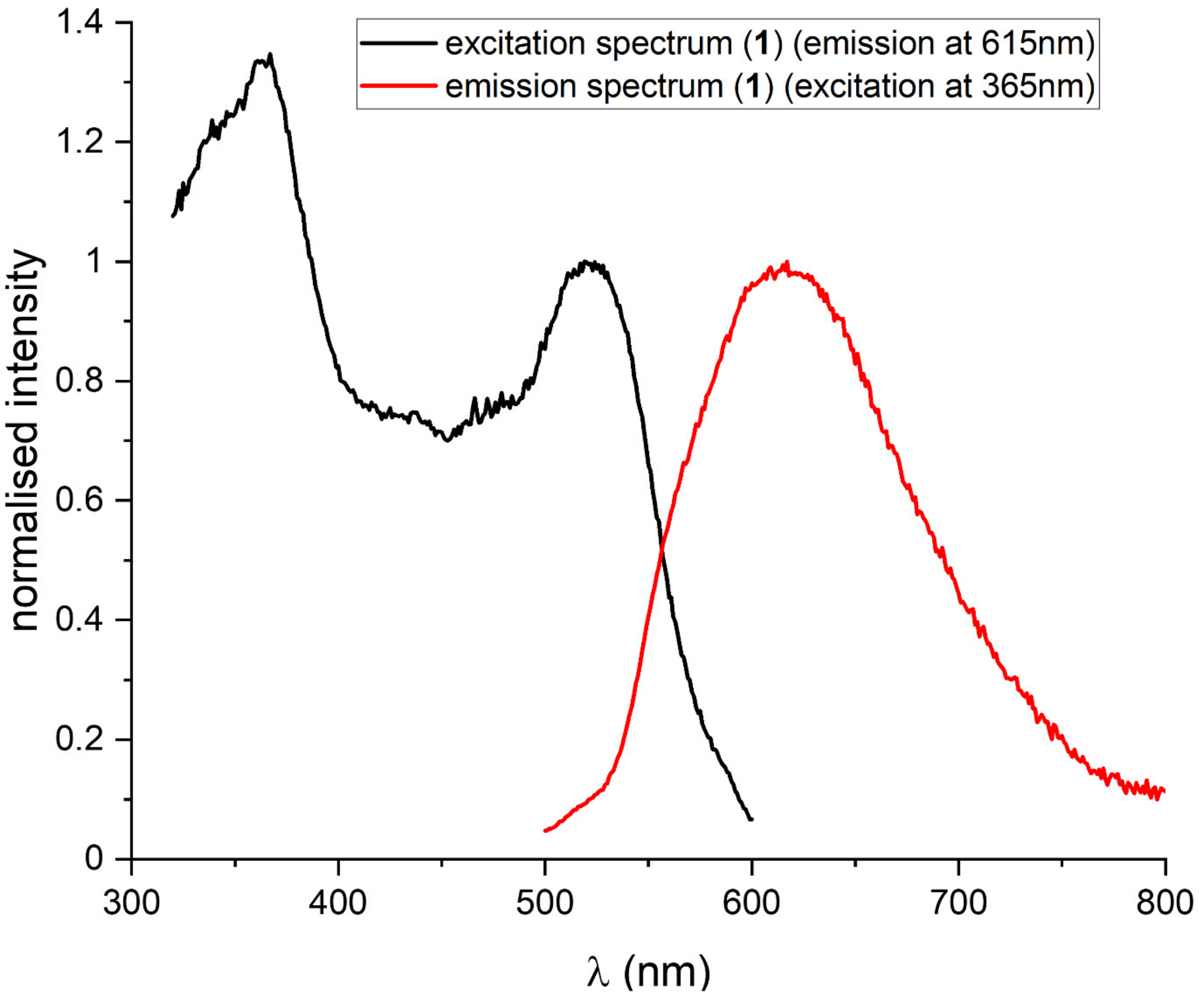

Figure S1. Solid state emission and excitation spectra of **1**. The peak at ca. 520 nm in the excitation spectrum indicates oxidation of **1** in line with the spectroelectrochemical data reported in the main text.

Transient Absorption Spectroscopy

The excitation into higher states was investigated using a 317 nm pump pulse. The results are similar to the dynamics observed for the excitation at 490 nm except for the GSB band which was previously observed at 420 nm. In the spectra after excitation at 317 nm shown in figure S2 below, the GSB only develops after *ca.* 1 ps. This is a result of initial VR from higher excited states into the S_1 state.

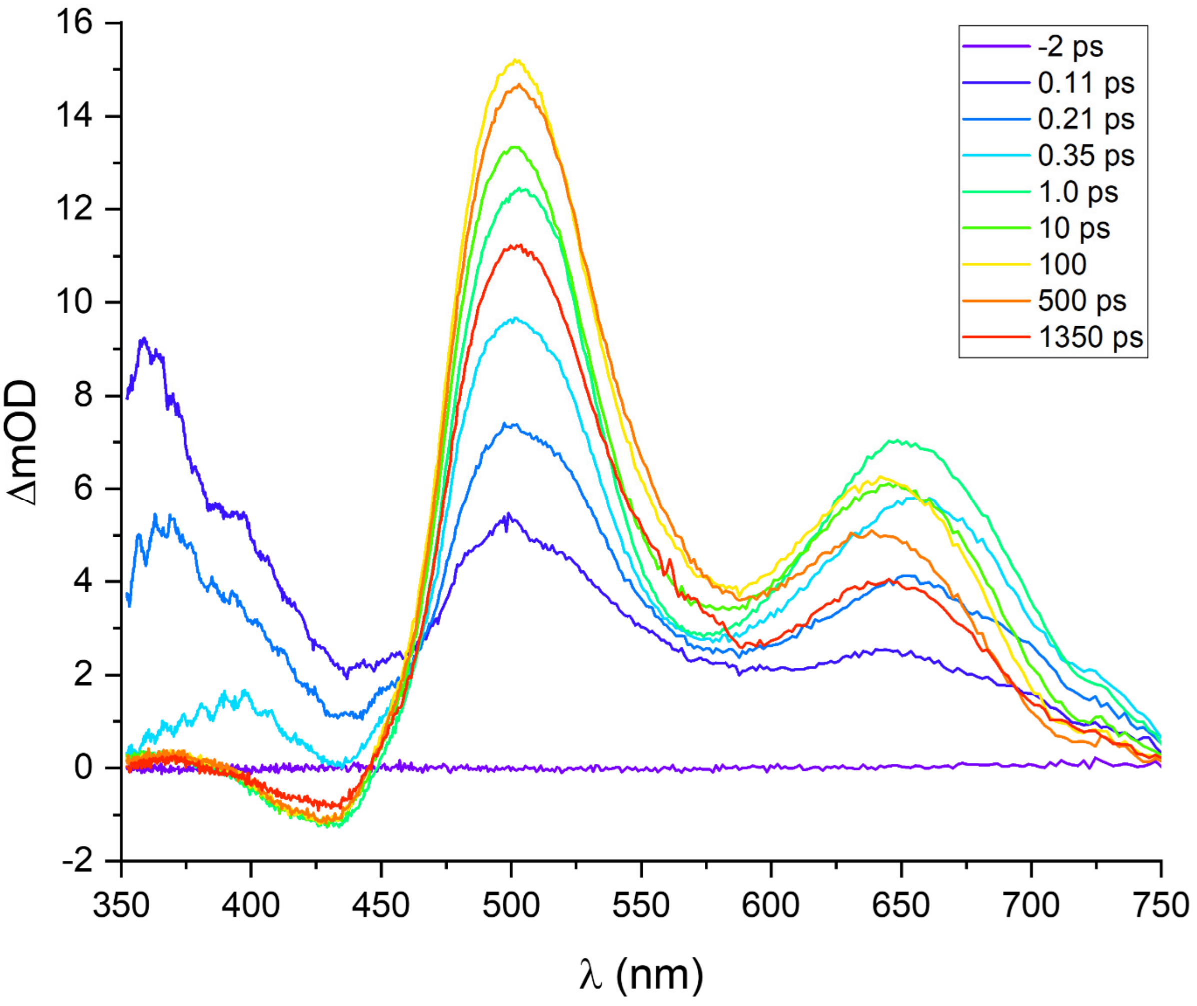

Figure S2. Transient absorption spectra of a fresh solution of **1** after excitation at 317 nm.

Further single transient analyses of the spectra recorded on a freshly prepared sample of **1** (Figure S3 a) as well as an aged solution of **1** (Figure S3 b) after an excitation with a 490 nm pump pulse were performed. The slow increase of both single transients at 530 nm indicates an IVR within the excited state or an energy transfer to the solvent. At 600 nm we observe a blue-shift of the absorption band in the aged solution which is completed after *ca.* 100 ps.

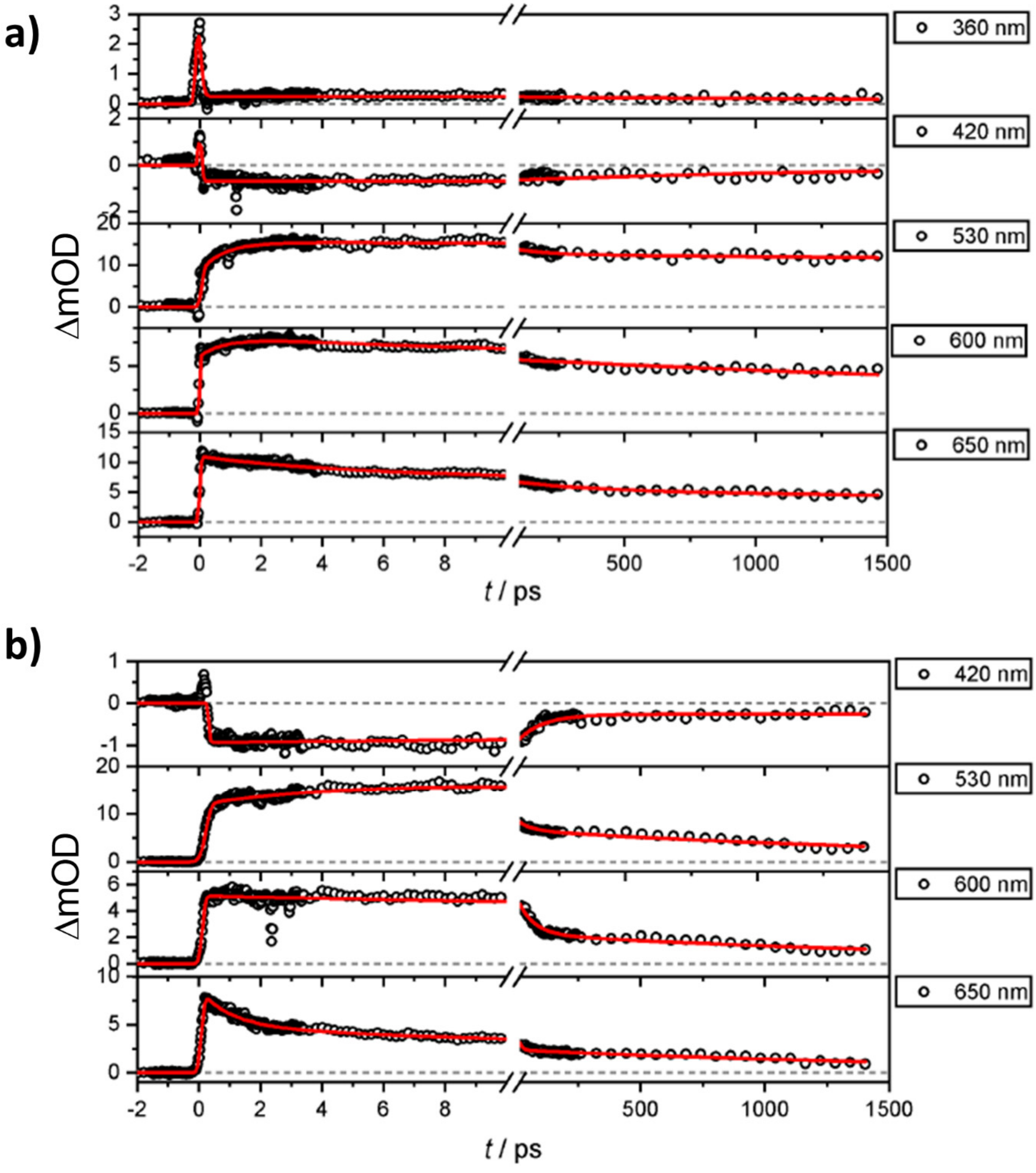

Figure S3. Single transient analyses of a fresh and an aged solution of **1**.

(Spectro)electrochemistry

Cyclic voltammetry was conducted on freshly prepared samples of **1** and **2** (see Figure S4). Several irreversible features were observed for both compounds.

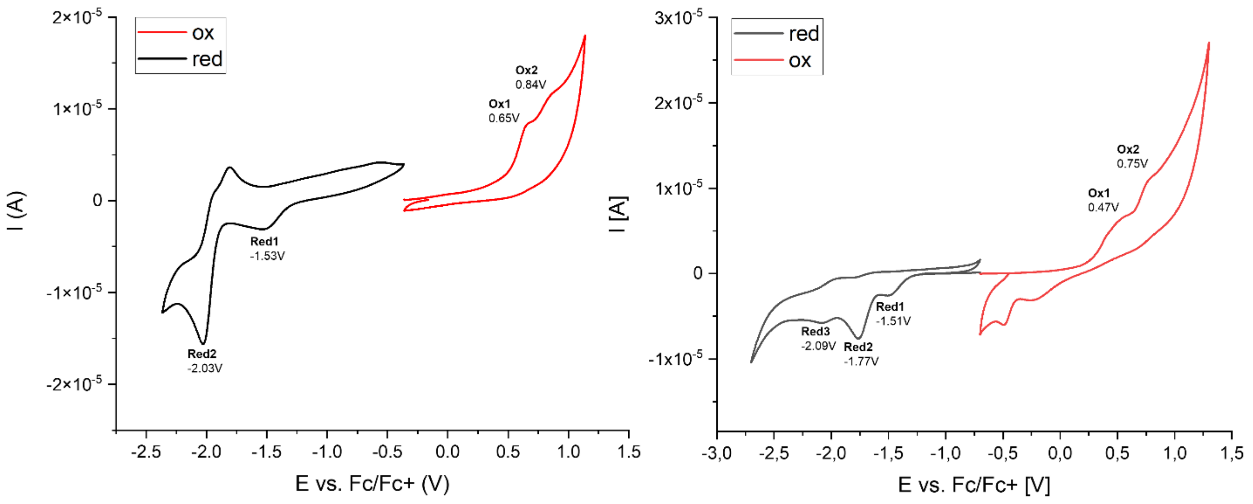

Figure S4. Cyclic voltammetry of **1** and **2** (left and right) in DMF with 0.1 M $TBAPF_6$ as electrolyte revealing the position of the first oxidation which is used for the spectroelectrochemical measurements.

Evolution of the absorption spectrum of **2** under an applied voltage of 0.7 V vs Fc/Fc^+ indicates the formation of an oxidised species reflecting the behaviour observed for compound **1**.

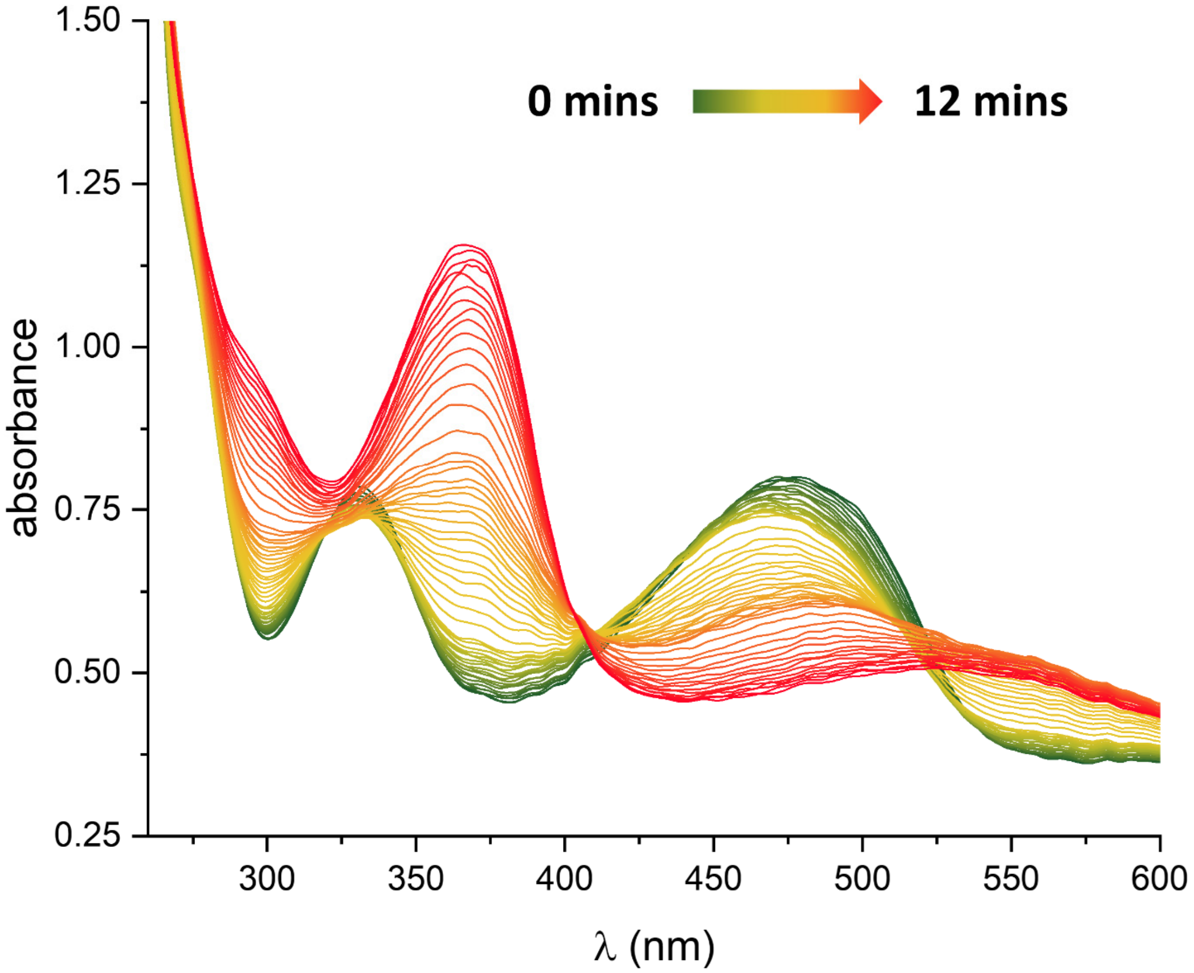

Figure S5. Spectroelectrochemical measurements of compound **2** at a voltage of 0.7 V vs Fc/Fc^{+} reveal the interconversion of the pristine compound (green) to an oxidised species (red) as suggested by the isosbestic points.

Following the decomposition of compound 1 using ¹H-NMR

The fresh spectrum (see Figure S6) of **1** was recorded approximately 10 mins after the start of the dissolution process since the compound does not dissolve easily in DMF and had to be stirred to reach a sufficient concentration.

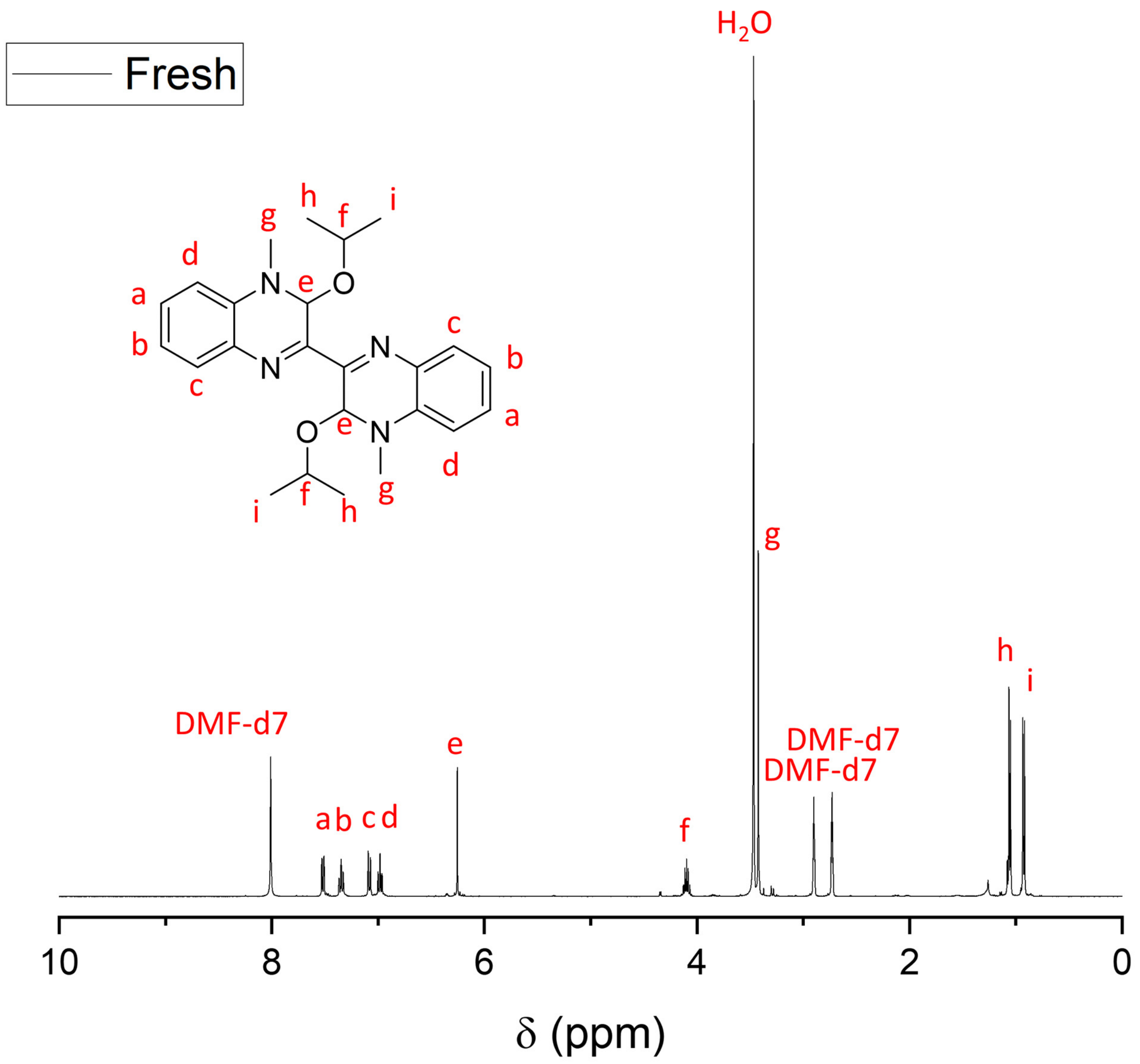

Figure S6. ¹H-NMR of compound **1**.

In order to describe the decomposition that **1** undergoes, a selection of spectra after several times after dissolution are shown in the following highlighting relevant sections of the NMR spectra (see Figures S7-S12).

After 1 hour and 15 minutes the decomposition can be observed in all four of the insets of Figure S8. In particular from the regions between 6.4-6.1 ppm and 4.4-3.7 ppm it furthermore becomes clear that multiple species are formed (see multiple sextets of the iso-propyl proton, green inset and the appearance

of multiple peaks in the blue inset). This trend progresses until after 26 h almost all of compound 1 has decomposed (see peak at 6.25 ppm and sextet centred at 4.1 ppm which are both essentially gone). From the appearance of peaks in similar regions such as the aromatic proton region in which, after 26 h, the multiplets overlap heavily, it can be concluded that the formed species possess similar structures. Moreover, the NMR signature of the starting material Mbqn-$(OH)_2$[1] can be identified amongst the newly formed species. In Figure S13 (reproduction of a figure published in the supplementary information of reference S1) several candidates for the newly formed species are shown which can form through equilibria described involving H_2O which is also present in the current NMR spectra.

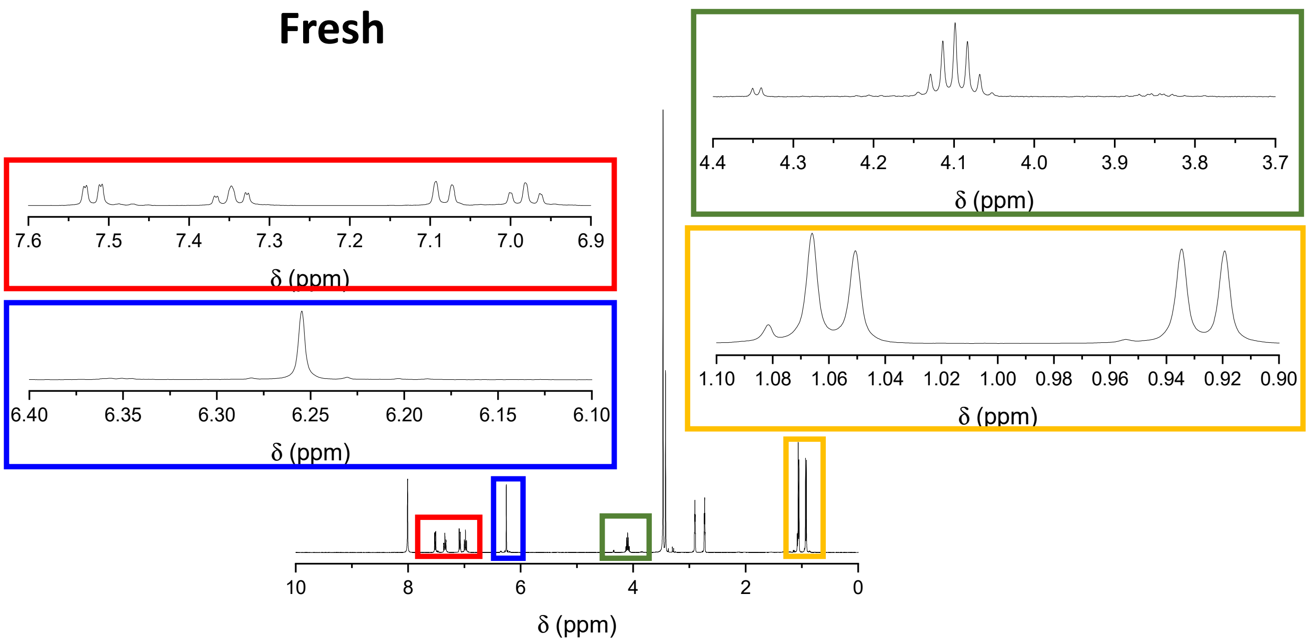

Figure S7. ^{1}H-NMR spectrum in DMF-d7 directly after the completed dissolution of **1**.

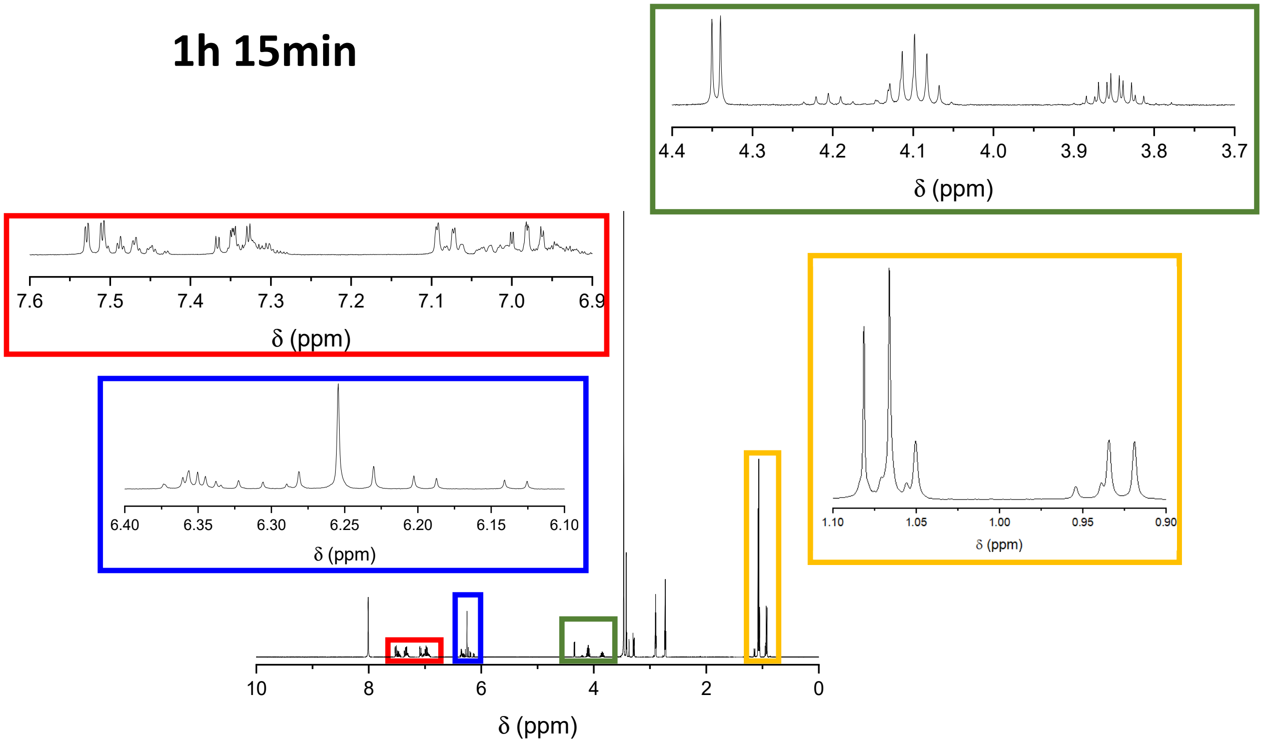

Figure S8. ^{1}H-NMR spectrum in DMF-d7 1 h and 15 min after the completed dissolution of **1**.

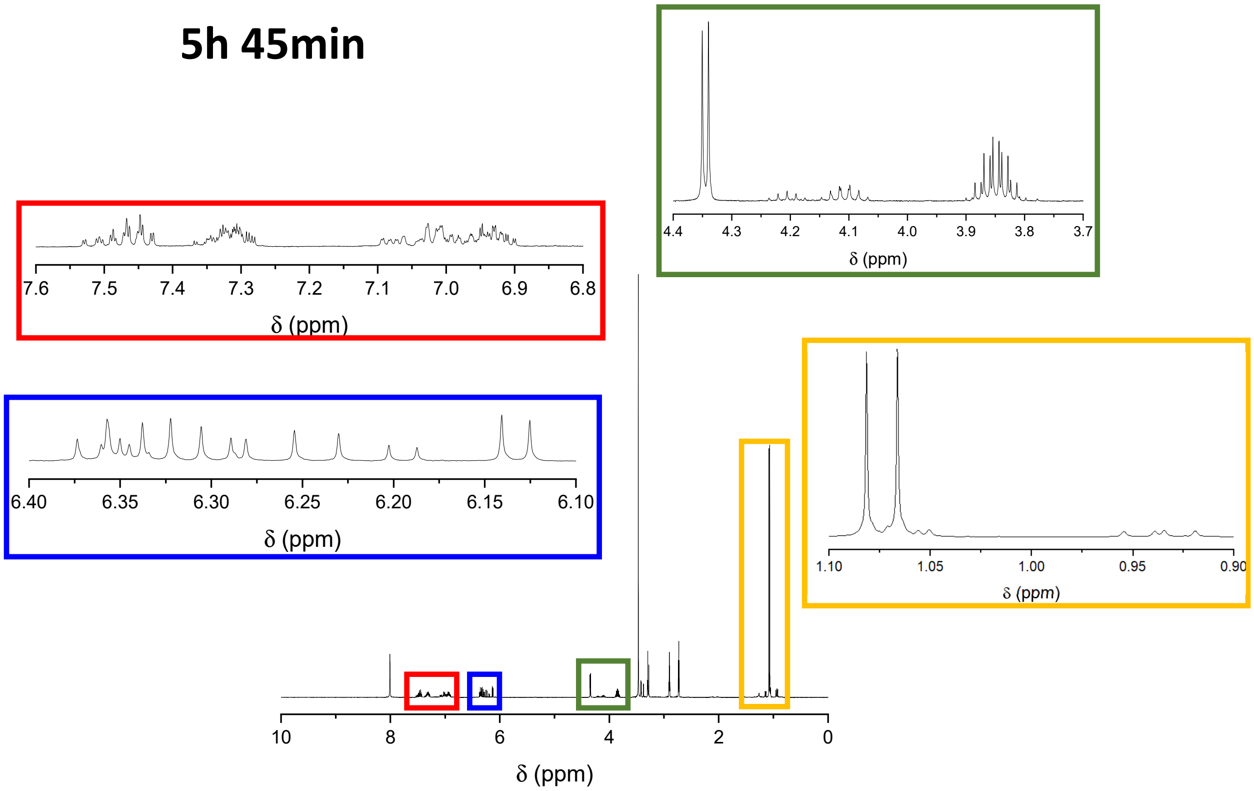

Figure S9. ^{1}H-NMR spectrum in DMF-d7 5 h and 45 min after the completed dissolution of **1**.

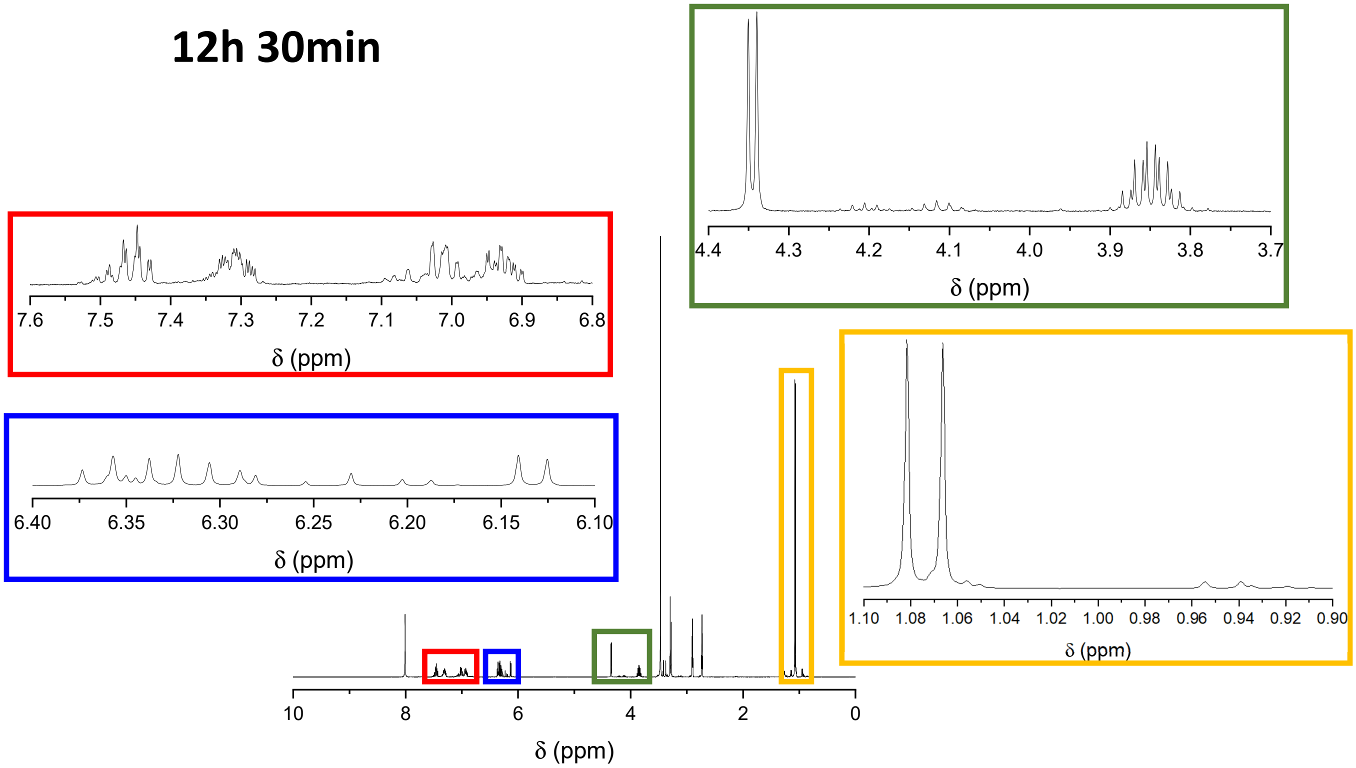

Figure S10. ^{1}H-NMR spectrum in DMF-d7 12 h and 30 min after the completed dissolution of **1**.

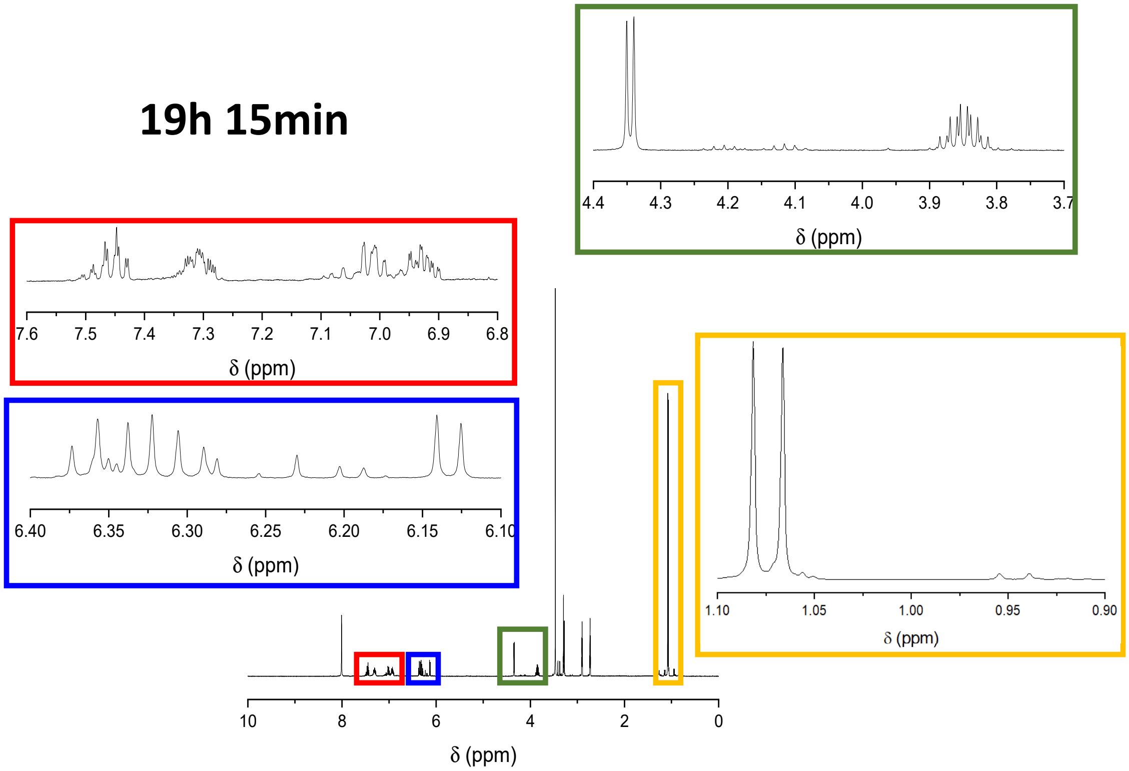

Figure S11. ^{1}H-NMR spectrum in DMF-d7 19 h and 15 min after the completed dissolution of **1**.

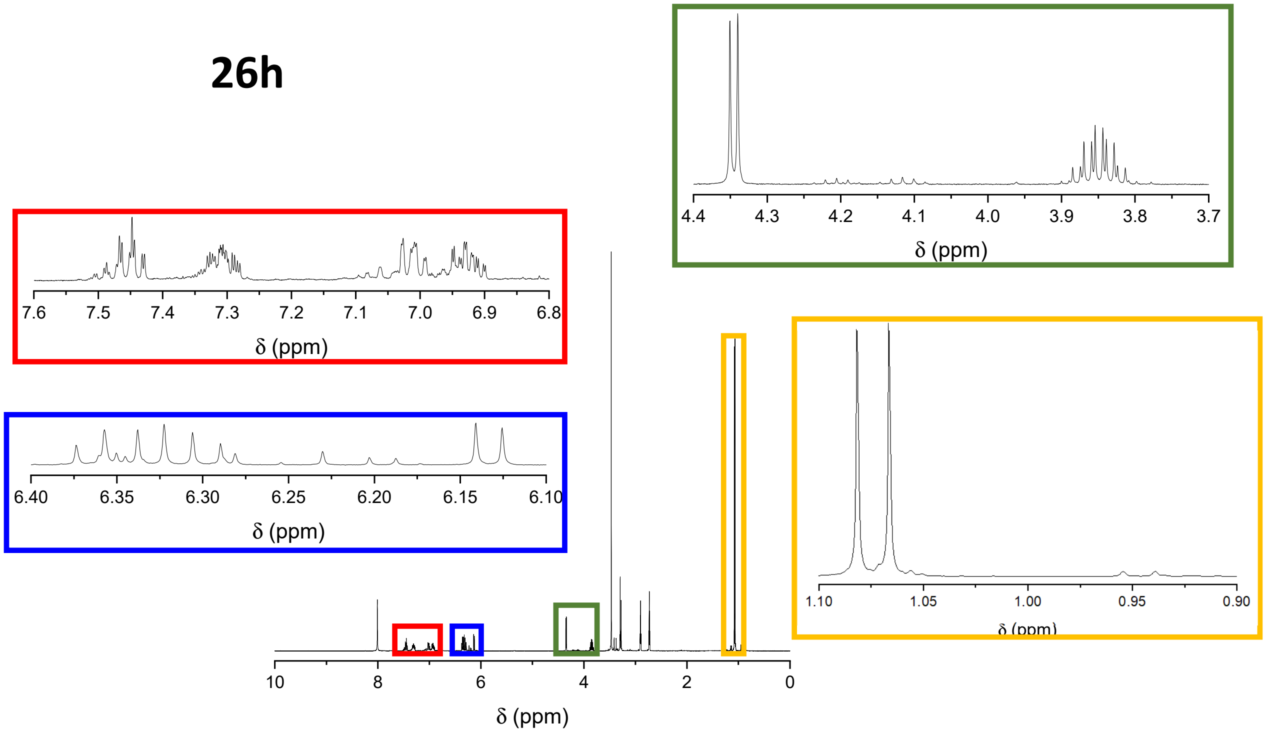

Figure S12. ^{1}H-NMR spectrum in DMF-d7 26 h after the completed dissolution of **1**.

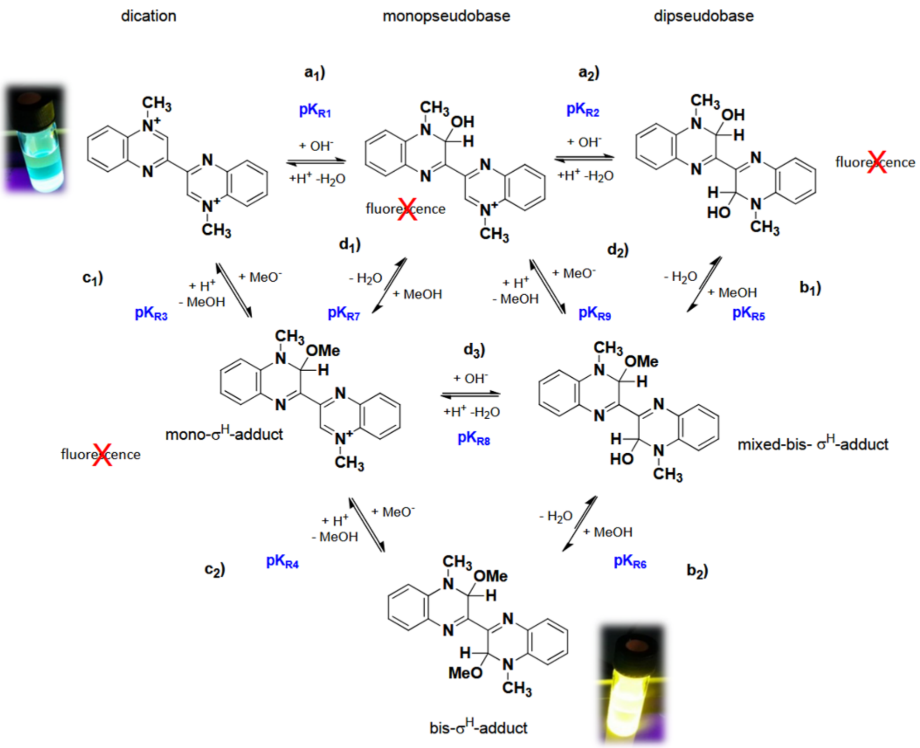

Figure S13. Several equilibria present in substituted biquinoxen systems resulting in different species, which are non-luminescent as observed during the decomposition of **1** (decreasing quantum yield).[1]

Figure S14 shows a normalized excerpt of the NMR spectra between freshly dissolved and 26 hours in the sensitive region including the iso-propyl sextet. From these we can conclude that significant decomposition of 1 occurs between 1 and 2 hours.

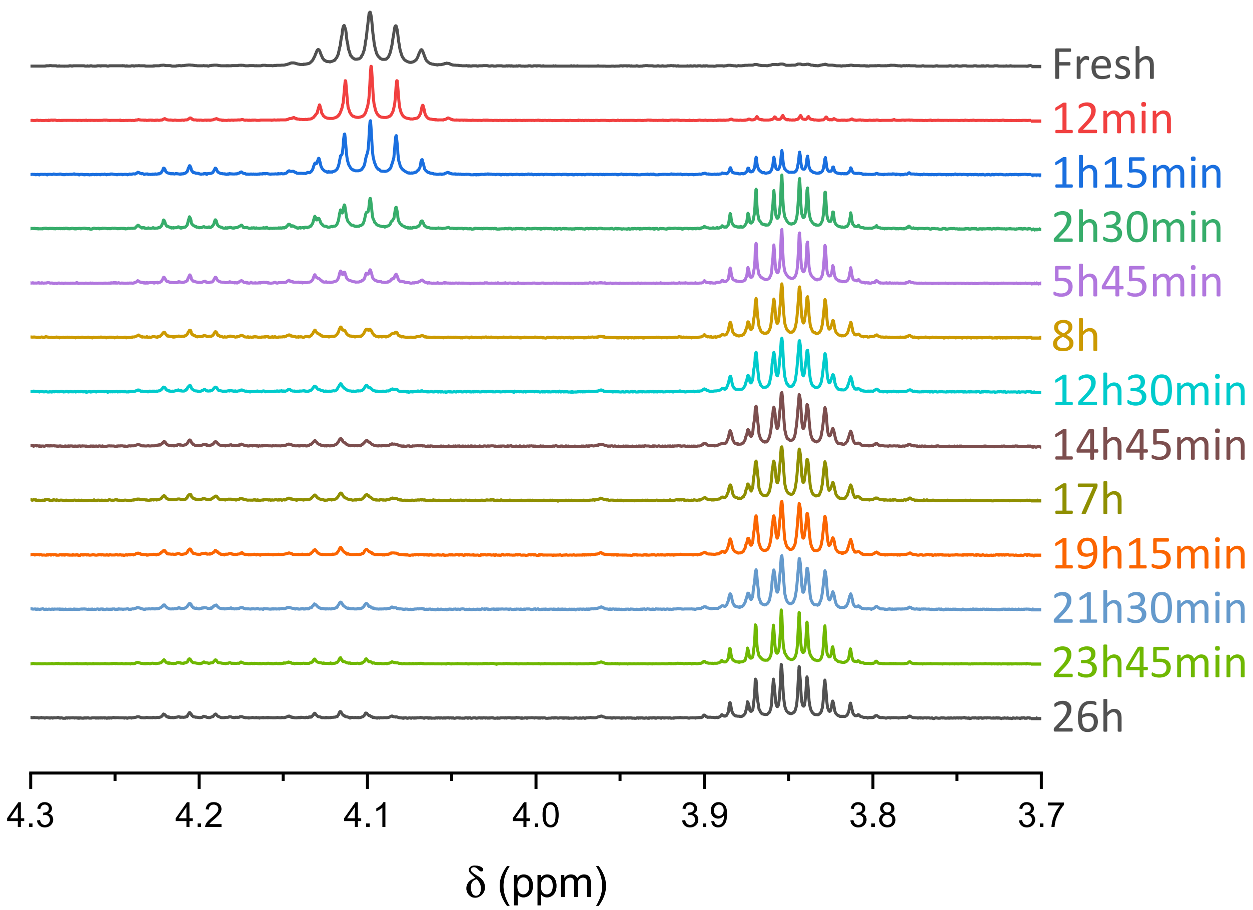

Figure S14. Normalized close-up of the iso-propyl sextet.

(1) Leblanc, N.; Genovese, D.; De Cola, L.; Powell, A. K. A platform with connections in many directions - further remarkable facets to the multifaceted methylbiquinoxen dication. *Phys. Chem. Chem. Phys.* **2017**, *19*, 6981-6988.
